# Census Bulletins, Nos. 24 to 60

**Published:** 1891-07

**Authors:** 


Census Bulletins, Nos. 24 to 60. Hon. Robert P. Porter, Superintendent of the Census, Washington, D. C.

These bulletins, issued regularly, have been received at this office, until now sixty have been published. We have noticed many of them from time to time, but have not space to give any extended summary. We select, therefore, those dealing with statistics of the Census of Education, from which it appears that Mississippi shows a gain of 47.90 per cent. in public school enrollment in the past decade. Louisiana has a 53.52 per cent. exhibit. Texas has gained 133.15 per cent.; North Carolina, 27.08; South Carolina, 50.89; Virginia, 55.06; West Virginia, 34.42. New Hampshire sustains a loss of 7.51 per cent., Maine 7.38, and Vermont one of 10.42 per cent. In the line of growth in public school enrollment Connecticut has a 6.68 per cent. gain; Massachusetts, 17.33; New York, 1.38; Ohio, 5.98; Pennsylvania, 1.59; Iowa, 15.88.

